# Adolescents’ own views on their risk behaviors, and the potential effects of being labeled as risk-takers: A commentary and review

**DOI:** 10.3389/fpsyg.2022.945775

**Published:** 2022-11-17

**Authors:** Ivy N. Defoe, Stephanie E. Rap, Daniel Romer

**Affiliations:** ^1^Forensic Child and Youth Care Sciences, University of Amsterdam, Amsterdam, Netherlands; ^2^Annenberg Public Policy Center, The University of Pennsylvania, Philadelphia, PA, United States

**Keywords:** adolescence, risk-taking, substance use, motivations, labeling effects, children‘s right to participation

## Abstract

Adolescents are stereotypically viewed as risk-takers (“stereotypical risk-takers”) in science, mainstream media, fictional literature and in everyday life. However, increasing research suggests that adolescents do not always engage in “heightened” risk-taking, and adolescents’ own perspectives (motives) on risk-taking are largely neglected in research. Hence, this paper is a commentary and review with two aims. First, taking a cross-national perspective, we discuss the definition of adolescence and risk behavior. We argue that much of the research on what drives adolescent risk behavior (e.g., substance use) focuses on the harms that this behavior promotes rather than on the need to explore and grow into adulthood. Thereafter we summarize the dominant approach to studying motives behind substance use, which has mostly considered young adults, and which has typically not focused on adolescents’ own self-generated motives. The few empirical studies (including one of our qualitative studies) on adolescents’ own motivations for engaging in risk behavior (i.e., cannabis use, alcohol use, and tobacco smoking) show that the most frequently mentioned motives by adolescents were being cool/tough, enjoyment, belonging, having fun and experimenting and coping. Interestingly, the “cool/tough identity” motive is virtually overlooked in research on adolescent risk-taking. The above-mentioned motives, however, generally support newer theories, such as the *Developmental Neuro-Ecological Risk-taking Model* (DNERM) and the *Life-span Wisdom Model* that suggest that adolescents’ motivations to engage in risk-taking include experimentation, identity development, explorative behavior, and sensation seeking, all of which run counter to the stereotype of adolescents engaging in risk-taking due to “storm and stress.” Hence, we also briefly consider additional recent attempts to study positive forms of risk taking. Second, extrapolating from sociological/criminological theories on labeling, we suggest that caution is warranted when (inaccurately) labeling adolescents as the “stereotypical risk-takers,” because this can instigate a risk-taking identity in adolescents and/or motivate them to associate with risk-taking peers, which could in turn lead to maladaptive forms of risk-taking. Empirical research testing these hypotheses is needed. To conclude we argue that research on adolescent risk-taking could further benefit from considering adolescent’s own motivations, which is also in line with the participatory approach advocated by international children’s rights standards.

## Introduction

### The ongoing question of what adolescence entails: A cross-national perspective

Adolescence is traditionally considered as the “in between” developmental phase, with the childhood phase at one end, and the adulthood phase at the other end. Being in this in-between phase, in which humans develop rapidly, has in part contributed to adolescence being described as a period of “storm and stress,” or a period of “confusion” (Hall, 1904). Adolescents have been thought of as being “stuck” in a tug of war between two distinct developmental phases—childhood and adulthood, struggling with their developing body and mind. At the same time, adolescence is clearly also an exciting period, filled with many opportunities, such as finding one’s identity and building a life of his/her own, less dependent on the guidance of their parents. What makes this period even more fascinating, is that there has never really been a consensus on which ages the adolescent phase encompasses. Nevertheless, many scholars, especially developmental scientists (e.g., Crone and Dahl, 2012; Steinberg et al., 2018; Defoe, 2021), posit that adolescence begins approximately at the start of puberty, around the ages 10–12, which is also the age when most youth in the Western-World transition from primary school to secondary school.

When exactly adolescence ends has been more subject to inconsistency and little consensus. According to some scholars becoming an adult can be viewed as a gradual process, this distinct phase in life has been referred to as “emerging adulthood” (Arnett, 2000). The definitions of adulthood in society are influenced by the cultural and normative framework on which the society is built. For example, in the Western world, the age when adolescence ends often coincides with when the laws of a country consider an individual as an “adult,” thus when individuals are allowed to attain adult roles in society. This has typically been the age of 18 or 21 (*cf* Shulman et al., 2016; see also article 1 UN Convention on the Rights of the Child (CRC)). Age 18 has additionally been the age when most have completed secondary school in the Western world, but also in the Caribbean and Latin America more generally, and it coincides with the age when people have the right to vote and when most countries try individuals as an adult in criminal court (i.e., the upper age limit of the youth justice system; see Cipriani, 2009). Interestingly, although age 18 corresponds with adult status in many Non-Western (e.g., the Caribbean) and Western (e.g., Western Europe) nations, individual cultures differ when it comes to whether this age is the appropriate age for sexual consent, marriage and child bearing (see for example Horii, 2021).

From a child development perspective, the age of 18 is rather arbitrarily chosen. Studies on human brain development indicate that the prefrontal cortex of the brain, which is involved in cognitive control, is not fully myelinated and pruned until the age of 24 (Giedd, 2010). Accordingly, some scientists (e.g., Crone and Dahl, 2012) argue that based on this biological marker, the definition of adolescence should be extended at least to the mid-twenties. The above-mentioned neuro-scientific findings have already impacted legislation. For instance, as of 2014, in the Netherlands young adults until the age of 23 can be imposed a youth justice sentence in criminal court, based on the personality of the accused or the circumstances under which the offense was committed (Matthews et al., 2018; Rap et al., 2020). In sum, when considering what adolescence entails, the culture(s) within a country is a decisive factor, and historically, culture impacts legislation (Defoe, 2021), which again in turn impacts how the span of the adolescent period is defined. The concept of adolescence can therefore be seen as a social construct that is influenced by the historical, cultural and societal context.

### The ongoing question of what risk-taking entails: A cross-national perspective

Closely related to the concept of adolescence is “risk-taking,” “risky decision making” or “risk behavior.” These terms focus either on the decision-making side (risky decision-making) or the behavioral side of risk-taking (risk behavior). For our purposes, we treat these interchangeably. Nevertheless, it is also noteworthy that risk-taking can be defined in different ways. From an economic perspective, typical for the decision-making sciences, the definition of “risk taking” is: *choosing an option with the most uncertain outcome* (Figner and Weber, 2011; Defoe et al., 2015). Alternatively, in (developmental) psychology, risk taking is described as *deciding to engage in behaviors that are associated with at least some probability of undesirable outcomes* (Boyer, 2006). Some psychological scientists have emphasized the cultural component of this definition. Namely, Defoe (2021) defined mal-adaptive risk behaviors (e.g., substance use and delinquency) as “*behaviors that are associated with some probability of a maladaptive outcome—that is an outcome that can impede the acquisition of culturally-accepted goals*” (Defoe, 2021; p. 2).

Of note is that despite the appearance of objectivity in the economic definition of risk-taking, the way in which researchers have studied this phenomenon has focused almost entirely on outcomes that are seen as undesirable from the adult’s perspective. For example, there are many behaviors children show that adults would find acceptable that also involve risks, such as learning a new skill (e.g., skiing), accepting an academic challenge (e.g., taking a more difficult course), or protecting a friend (e.g., intervening to stop a bully). But hardly any of the research on adolescent risk-taking has examined these “conventional” risks, which presumably also increase during the adolescent period (Romer et al., 2017; Defoe and Romer, 2022). Moreover, in recent years increased attention has been directed toward participatory forms of research and research that focusses on the views and perspectives of children and young people, in line with their right to be heard and to participate (article 12 CRC), in order to better understand their lifeworld from their point of view (for an overview see Sommer et al., 2021; Freire et al., 2022).

No matter the definition of risk-taking, behaviors such as substance use and delinquency are often the focus of (maladaptive) risk behaviors in adolescence (Defoe and Romer, 2022). In this paper we focus on substance use because it is a widely studied risk behavior that rises rapidly during adolescence in many cultures (Willoughby et al., 2021) and is among the most studied risk behaviors in the literature. These risk behaviors typically show accelerated increases during adolescence and/or emerging adulthood (Steinberg, 2015). In fact, adolescents are stereotypically viewed as risk-takers (“stereotypical risk-takers”) in science, the mainstream media, fictional literature and in everyday life. This goes as far back as over 400 years ago when Shakespeare described the ages between 13 and 20 as a period of heightened levels of misbehavior (e.g., stealing, and substance misuse; Shakespeare, 1623). In science, this characterization of adolescence has contributed to the formulation of multiple impactful developmental theories that aim to explain why adolescents engage in higher levels of risks (i.e., “heightened adolescent risk-taking”) compared to children and adults. In society, this has contributed to negatively labeling adolescents (see more below).

#### New directions toward risk-taking

We start from the presumption that there are at least two issues with the current conceptualizations of adolescence, risk-taking and the associated communication about this developmental phase. First, in this article we argue that in order to achieve a more comprehensive understanding of risk-taking during adolescence, the perspectives and experiences of the adolescents themselves need to be identified. That is, which behaviors do *adolescents* define as risky, and which motivations do *adolescents* report for engaging in those behaviors? In a unique recent qualitative study (*N* = 57; 77% female; >90% European American), adolescents were asked the following question “What behaviors come to mind when I say “risk behavior?” (Skaar, 2021). The findings revealed that educational risk behaviors (e.g., taking challenging classes) were the most mentioned by youth as risk behaviors, followed by drug and alcohol use and “trying something new.” Behaviors that were mentioned less often were “standing up to bullying,” “out of comfort zone” and “alcohol use” (Skaar, 2021). Although the fact that educational risk behaviors being the most mentioned, was perhaps an artifact of the convenience sample that was used (i.e., students who were enrolled in an Advanced Placement Psychology course; *cf* Skaar, 2021), it is still telling that this rarely studied “risk” was mentioned by far the most by youth, whereas alcohol and drug use were the second and third runner up (Skaar, 2021). When it comes to cross-national differences, such studies are rare, but it has been reported that youth in Turkey report substantially different risk behaviors, such as wearing revealing clothes, engaging in political activism, and losing one’s virginity, compared to youth in countries such as the United States (Kloep et al., 2009). The, adolescents’ perspectives on what risk-behaviors entail might be very different across countries, again influenced by the cultural norms in which they grow up. Hence, in this review, we focus primarily on adolescents’ motives for engaging in the most common substance use behaviors during adolescence, namely, alcohol use, cannabis use and tobacco use (smoking). Unfortunately, only few developmental theories on risk-taking consider adolescents’ perspectives.

In this review we will discuss two relevant theories, namely the Lifespan Wisdom Model (Romer et al., 2017) and the Developmental Neuro-ecological Risk-taking Model (DNERM; Defoe, 2021). The gap in theoretical substantiation is in turn also reflected in the few empirical studies that have investigated this phenomenon. Most studies on this topic have focused on emerging adults (ages 18–25; often college students; for reviews see: Cooper et al., 2015; Votaw and Wikiewitz, 2021). However, motivations might differ across age, which makes it pertinent to investigate adolescent specific motivations (*cf* Cooper et al., 2015). Also, the legality of using such substances as alcohol, cannabis and tobacco depends on the age of the user (i.e., the adult status of the user), and thus youth might have different motives to use substances than adults.

As far as we know, this paper is the first to review the few empirical studies on adolescents’ own motivations for engaging in substance use (cannabis use, alcohol use, and smoking). In doing so, we use the state-of-art review of Cooper et al. (2015) as a point of departure, and we also draw conclusions from a more recent review that focused specifically on ecological momentary assessments (Votaw and Wikiewitz, 2021). Moreover, we will review findings from a qualitative study by one of the authors (see Defoe et al., 2016; Lochs, 2020; Tabor, 2020), which is one of few studies that examined self-generated motives by adolescents in the Netherlands for engaging in risk behavior.

Second, increasing research suggests that adolescents do not always engage in “heightened” risk-taking (for a meta-analysis of experiments, see: Defoe et al., 2015; and a review of real-world risk-taking behaviors, see: Willoughby et al., 2021). Yet adolescents are widely labeled as the stereotypical risk-takers, while investigation of possible consequences of such an ingrained (and inaccurate) label on adolescents’ actual risk-taking appears to be uncharted territory. Therefore, we explore the *labeling theory* (Becker, 1963) as a framework to discuss why such often-used conceptualizations of the adolescent period can be counter-productive when not used thoughtfully. Finally, we consider implications for policy.

## Two recent theories that consider adolescent’s motivations for engaging in risk behavior

### Life span wisdom model

Before focusing on adolescents’ own motives for engaging in risk behavior, it is of relevance to briefly highlight the two previously mentioned developmental theories that explain risk taking behavior in adolescence while considering adolescents’ perspective. The first theory is the Lifespan Wisdom Model (LSWM; Romer et al., 2017) that considers adolescent motivations for novelty seeking. This model focuses on the adaptive function of sensation seeking during adolescence as a process that encourages exposure to novel experiences that can further the development of wisdom. Wisdom has traditionally had many meanings (Curnow, 2015), but consistent across those interpretations is the accumulation of experience that allows one to make prudent decisions. Trying a substance like alcohol could be one step in this direction as far as this widely used beverage is concerned. It is nevertheless unfortunate that some adolescents will over-use the substance leading to a disorder. In other cases, such as use while driving, it may also cause harm. Society recognizes these problems and tries to discourage their occurrence, by limiting the sale and distribution of alcohol to youth.

According to the LSWM, some adolescents with high sensation seeking tendencies also have high levels of impulsive behavior tendencies, which reflect weaker abilities to refrain from immediately rewarding experiences, such as drug use. There is evidence that this tendency increases during adolescence for those with higher levels of impulsivity, thereby predisposing to continued use of drugs and risk for addiction (Khurana et al., 2018; Khurana and Romer, 2021; Khurana et al., 2022). Such youth also have difficulties in learning from experience because of their heavier attraction to immediate reward, which also increases the risks for addiction and other problem behaviors (Khurana and Romer, 2021). Nevertheless, the LSWM does not attribute all adolescent risk taking to a deficit in cognitive control relative to sensation seeking, since the two processes tend to increase in tandem as adolescents age. In addition, it is primarily older adolescents and young adults who engage in what is regarded as maladaptive “real-world” risk behaviors, due to greater opportunities for such behavior (Willoughby et al., 2021; see also Defoe, 2021). This is in contrast to what is predicted by brain imbalance models which predict greater risk taking during mid-adolescence when the imbalance should be greatest (see, e.g., Casey et al., 2008; Steinberg, 2008).

### The developmental neuro-ecological risk-taking model

The second theory we highlight is the Developmental Neuro-Ecological Risk-taking Model (DNERM), which hypothesizes that developmental phase (age) and culture predict levels of risk exposure (i.e., exposure to risk conducive situations; Defoe, 2021). That is, multiple types of risk exposures increase with age (at least up until young adulthood), and hence the developmental increases in risk behaviors we observe in the real-world. Also, certain types of risk behaviors would be more common in cultures where they are accepted (e.g., alcohol has been typically culturally accepted in the Western World). In the event of (physical) risk exposure, DNERM hypothesizes that particularly younger youth (versus older youth) will be more likely to engage in heightened levels of risk-taking *via* cue reactivity mechanisms and their natural tendency to explore their surroundings (Defoe, 2021). However, this link from risk exposure to risk behavior could further be moderated by youth’s cognitive and affective self-control (perhaps especially affective self-control; Defoe, 2021). In other words, in relation to motivation, DNERM suggests that physical risk exposure in itself predicts potentially maladaptive risk behavior (e.g., substance use and delinquency), due to the curiosity and desire that a risk conducive situation can elicit, especially for youth (Defoe, 2021). That is, risk exposure in the context of youth is associated with their need for exploration, which explains why a novel risk exposure is attractive for the adolescent who is still exploring his/her identity, and experimenting to learn if one fits with his/her surroundings (see, e.g., Erikson, 1968).

Indeed, as will be seen below in our qualitative study, perhaps adolescents, quest for especially a “cool/tough identity” is important to understand their engagement in risk behavior, since in that study (Lochs, 2020; Tabor, 2020; see also Lee et al., 2007), adolescents mentioned being cool/tough as the primary motive for engagement in alcohol, tobacco, and cannabis use. However, according to DNERM, whether this curiosity for experimenting and exploring one’s identity will result in substance use (and other potentially maladaptive risk behaviors) could further potentially depend on self-control—an interaction effect which remains to be investigated in research.

The above-described theories place more emphasis on the exploratory motives for such risk behaviors as substance use, an area of research that has received far less attention in the literature than a focus on motives for regular use of substances. However, recent work has begun to explore the factors that encourage what has become known as positive risk taking (Duell and Steinberg, 2021). We examine this new direction after we review the much larger volume of research that has dominated the field.

As mentioned earlier, the most comprehensive model of motives for substance use is due to Cooper et al. (2015). Their model divides these motives into two dimensions: internal vs. external and approach vs. avoidance. Internal avoidance motives entail the effort to cope with negative emotions while external avoidance involves conformity pressures to use drugs. Internal approach motives involve feelings of enhancement due to the drug, while external approach motives involve socializing and the use of substances to facilitate those interactions. In essence, this is thus a two-dimensional model of motivation for substance use (Cooper et al., 2015). The motives are typically measured using ratings of outcome expectancies for the use of a drug in general or in a specific situation. It is also noteworthy that most of the research assessing these constructs involves either college undergraduates or adult community members. As a result, what is learned in this research is more applicable to established users of substances rather than adolescents who are just starting to experiment with substances.

## Adolescents’ motivations for engaging in risk behavior

Variations in motives for substance use are important to consider as they have predicted the frequency, quantity and extent of problems associated with substance use (see Cooper et al., 2015 for an overview). In the current review, we first focus on the motivations behind youths’ alcohol, cannabis and tobacco use as they have been described in past research. Adolescents’ motivations for engaging in substance use have successfully been incorporated in treatments, for example to lower heavy cannabis use among adolescents (Blevins et al., 2016). Some research has examined the motivation underlying the use of all three substances in adolescents. For example, Hansen et al. (2022) summarized 25 studies that assessed use of all three substances by adolescents. They found that across all three substances, peer use and injunctive norms for use were important predictors. The valence of attitudes and beliefs about the consequences of substance use were also important. However, parent perceptions were much less predictive. Despite this large compendium of studies, we learn very little about the motivation for use of these substances from this work other than that what peers are seen as doing is important in the lives of adolescents.

Another review summarized the findings from 64 studies using ecological momentary assessment, mostly with non-Hispanic White college students (Votaw and Wikiewitz, 2021). This research found that conformity motives were seldom studied and when they were, participants rarely reported this motive. The most frequent motives fell under the internal enhancement category in Cooper et al.’s scheme. Socializing was also frequently mentioned along with coping. Thus, one primarily gets a picture of motives for established use of these substances from this work.

A widely known motivational model for substance use is the alcohol motivational model of Cox and Klinger (1988, 1990, 2004), which is also used as the theoretical framework in the above-mentioned review of Cooper et al. (2015). It was initially developed for alcohol use, but paved the way for understanding motivations for the use of other substances as well, such as tobacco and cannabis (Cooper et al., 2015). Additionally, an extensive review demonstrated that this motivational model is generally applicable to tobacco and cannabis use by (emerging) adults (see Cooper et al., 2015). Although the review by Cooper et al. (2015) did not specifically focus on youth, the authors noted that motivations may differ across developmental stages of a person. For example, some studies have found identity motives to be specific for youth (Cooper et al., 2015), perhaps because adolescence is a period of significant identity formation which could ignite curiosity to experiment with substances (Defoe, 2021). Along those lines, youth who have not tried substances as yet, might have substantially different motivations for substance use compared to adults. For example, considering that adults have more experience using any drug, they are expected to be less motivated by curiosity compared to adolescents. Hence, previous literature that has especially focused on adults might not provide the most accurate representation of motives for adolescents.

The aformentioned Cooper model can be contrasted with the motives that are mentioned most by youth themselves. In our above-mentioned qualitative study (see Defoe et al., 2016; Lochs, 2020; Tabor, 2020) among 582 ethnically and socio-economically diverse Dutch youth (45.40% female; ages 13–16), who participated in a second wave data-collection of a 3-wave longitudinal study (for details on the sample, see Defoe et al., 2016), adolescents’ own perspectives on reasons for engaging in risk behavior were assessed. In that study the adolescents were asked to think of reasons why they or other youth drink large quantities of alcohol, smoke tobacco (cigarettes), and/or use soft-drugs (cannabis). A subsample of the youth answered these questions for alcohol (*n* = 360), smoking (*n* = 361) and cannabis use (*n* = 389). Of the answers given to these open-ended survey questions, the “being or acting cool/tough” motive (“stoer doen”) was reported the most for alcohol, cannabis and tobacco use (Lochs, 2020; Tabor, 2020). Interestingly, this “cool identity” motive did not emerge in the adult literature on motives, which was typically based on closed-ended survey questions, but it did emerge in literature on youth cannabis use when open-ended questions were used, although it was mentioned to a much lesser extent than other motives (see: Lee et al., 2007). Perhaps the first publication to systematically investigate the meaning of coolness consisted of a series of three studies which were conducted primarily among North American (United States and Canada) university students. In that study, the participants described “coolness” with socially desirable attributes (e.g., social, popular, talented). Additionally, factor analyses identified two factors for coolness. Namely the first factor “Cachet coolness” reflected active, status-promoting, socially desirable characteristics (Dar-Nimrod et al., 2012, 2018). The second factor “Contrarian coolness” reflected rebellious, rough, and emotionally-controlled characteristics. The authors concluded “*coolness is reducible to two conceptually coherent and distinct personality orientations: one outward focused and attuned to external valuations, the other more independent, rebellious, and countercultural*” (Dar-Nimrod et al., 2012; p. 175). A follow-up study (Dar-Nimrod et al., 2018) based on university students in Canada (17–36 years; *M* = 19.91; *SD* = 2.92) largely replicated these findings and additionally showed that the coolness concept is not captured in the Big Five personality characteristics. Of note, is that Contrarian coolness is what the Dutch youth appear to be referring to with the phrase “stoer doen” (“acting cool/tough”) in the above-mentioned qualitive study (Lochs, 2020; Tabor, 2020). However, it is still questionable to what extent the abovementioned findings (Dar-Nimrod et al., 2012, 2018) that are primarily based on university students would fully generalize to adolescents, but they could provide a starting-point for such research on adolescents.

The second most reported motive among Dutch adolescents for alcohol, cannabis and tobacco use, was a self-focused motive, namely “enjoyment” [e.g., it is tasty (“lekker” in Dutch)]. Other motives that were frequently reported were all self-focused motives, namely experimenting, stress reduction, and having fun (Lochs, 2020). Addiction was additionally frequently mentioned as a motive for tobacco use (Tabor, 2020).

Three important conclusions can be drawn from the above-mentioned findings based on Dutch youth. First, youth mention both self-focused and social-focused motives for engaging in substance use, and hence although studies suggest that substance use in adolescents is primarily a social behavior (e.g., Roditis et al., 2016), the notion that the main factor why youth engage in substance use is due to peer influence (e.g., because their peers are doing it, or because they feel pressured from their friends) is not entirely true. Also, even when the youth reported that they engage in substance use because their peers are doing it, negative forms of peer influence such a peer pressure was rarely mentioned, although especially such negative conceptualizations of peer influence are most common in the literature (*cf* Defoe et al., 2018).

Secondly, the most frequently mentioned motive by youth “being cool/tough” is not a common factor that is investigated in adolescent risk-taking research. Of note is that “being cool” which has been conceptualized as “image enhancement” (Lee et al., 2007) or as “impressing others” (conformity motive) in the motivation literature (see, e.g., Lee et al., 2009) may be a different form of social influence than social pressure which is a common theme in the adolescent risk-taking literature. It would be of added value for future research to look into what “being cool” essentially means from an *adolescent’s perspective*, as surprisingly, such research does not exist in the risk-taking literature, to our knowledge.

Thirdly, as the second motive for all substance use, youth mentioned the enjoyment that they experience while using substances, for example due to the feeling they receive from the substance or due to its taste. This sensory motive is virtually absent from both past and current theories on adolescent risk-taking.

Although being cool/tough was by far the most frequently mentioned motive for alcohol, smoking and cannabis use among Dutch adolescents, research on motives that were primarily based on adults and college students show that motives can differ across substance use type (Cooper et al., 2015). Moreover, three other reasons for doing so can be mentioned as well. First, the three substances have different psychoactive effects, and thus different susceptibility for addiction as well. Nicotine is most addictive of the three (Rigter, 2020). Secondly, whereas alcohol has been classified primarily as a depressant, cannabis has been primarily classified as a hallucinogen and nicotine as a stimulant, which may affect the motivations for using a certain substance (Rigter, 2020). Thirdly, the availability of these substances can differ dramatically. For example, although in most Western and non-Western countries, both alcohol use and smoking is legal for persons ages 18–21 and over, recreational cannabis use is illegal for all ages in most countries, although cannabis (especially medical cannabis) is increasingly being legalized. Thus, since recreational cannabis is illegal for persons below 18 or 21, it would be expected to be most difficult to acquire, perhaps more so for youth below those ages. Relatedly, another reason for considering the motivations for the use of these substances separately, is the cultural acceptance of substances. An example is the Caribbean island of Sint Maarten, where alcohol and tobacco are available to a similar extent, but still culturally alcohol use is more accepted than tobacco use (Defoe, 2021). The cultural acceptance can differ cross-nationally too. For example, generally speaking, in the Middle East, alcohol is less culturally accepted than in the Caribbean, and hence it is to be expected that alcohol use in the Caribbean would be more common. Hence, taking into account the possibility that motives can differ across substances, below we summarize the literature separately for youth’s motives for engaging in alcohol use, tobacco use and cannabis use.

### Current descriptions of adolescents’ motivations for engaging in alcohol use

As with any risk behavior that emerges in adolescence, the focus has been on the maladaptive aspects of the behavior. In the case of alcohol, there has long been the concern that alcohol use in adolescence will lead to alcohol use disorder later in life (Grant et al., 2001). There is also the concern that it will impair activities such as driving (Hingson et al., 2009). Both concerns are valid, but it is also important to recognize that alcohol is the most used substance in many parts of the world, and that for example most adults in the United States have used it at some time as part of a social gathering or source of relaxation (Cooper et al., 2015).

Alcohol is a sedative which means that it can reduce anxiety and make one feel relaxed (Wenk et al., 2017). For some, it can also produce euphoric effects, all of which can be attractive to adolescents. As a result, there are many reasons why adolescents might become interested in trying alcohol. Of note is that adolescents who are prone to dependence on alcohol are also more likely to experience internalizing symptoms (Deas-Nesmith et al., 1998). Those youth may well be using alcohol for its sedative effects. In our above-mentioned study among Dutch adolescents, primarily “being cool/tough” was mentioned by far the most, enjoyment (i.e., for the “taste,” for the feeling”) was 2nd runner up, followed by to be cozy (gezellig in Dutch), “for fun,” and “to belong,” which were all mentioned a similar number of times (Tabor, 2020). Thus, social conformity and sensation seeking (“for fun”) motives that are common in the literature are often mentioned by youth, although youth mentioned “being cool/tough” and enjoyment (sensory) motives more often whereas these motives are not typically considered in adolescent risk-taking research. Hence, it would be for example interesting for research to investigate whether the motives coolness and enjoyment predict adolescent substance use to a similar extent as more often investigated factors such as peer influence and sensation seeking,

As mentioned above, the research literature over the years has focused on social influences, with strong evidence that both family (Donovan, 2004) and peer use (Leung et al., 2011) are associated with greater likelihood of trying alcohol as well as tobacco and cannabis (Marziali et al., 2022). There is also strong evidence that advertising for alcohol on television and in magazines encourages adolescent trial (Smith and Foxcroft, 2009).

Much research has also focused on personal characteristics that predispose to alcohol use in adolescence. This research tends to find the same predictors as for use of other substances, like tobacco and cannabis. Youth with higher levels of sensation seeking as mediated by expectancies for alcohol’s positive affective effects have been found to try using alcohol and other substances before others do (Romer and Hennessy, 2007). It is less clear however that this characteristic is predictive of alcohol use disorder (Khurana et al., 2018). In any case, it is informative to examine the role that sensation seeking plays in predisposing interest in substances such as alcohol. Sensation seekers are open to trying new experiences and this extends to the use of substances. However, sensation seeking increases during adolescence, suggesting that this drive is biologically based in the dopamine reward system, which is attuned to novel reinforcing experience (Khurana et al., 2018). It would seem therefore that interest in trying a substance like alcohol would be expected, especially given its widespread use among adults.

In sum, alcohol use by adolescents is likely motivated by its widespread use by adults which makes it appear more acceptable as a substance and also more available in the home and elsewhere. Youth with greater exploratory drives, such as sensation seeking, are also more likely to try alcohol at an early age and if peers and family use the substance, this will make it all the more socially acceptable and “cool” to the adolescent.

### Current descriptions of adolescents’ motivations for engaging in smoking

Another risk behavior among adolescents is smoking cigarettes and recently the use of e-cigarettes (also known as vaping). Smoking tobacco differs however crucially from drinking alcohol or using cannabis. It does not cause disabling states of intoxication, such as hallucination, it improves working memory and concentration and suppresses appetite, it is much more addictive and hence withdrawal symptoms set in quickly (Cooper et al., 2015). These characteristics of smoking tobacco make it more likely that people are motivated to smoke more frequently, for a larger number of purposes and in a variety of daily situations (Cooper et al., 2015).

Research has predominately focused on personal and demographic factors predicting smoking initiation. Several factors have been found to be associated with ever and current cigarette smoking among youth, such as having parents or friends who smoke, the likelihood of accepting a cigarette from a friend, academic success, other substance use, sensation seeking and friends’ attitudes (Khuder et al., 2008; Guo et al., 2013; Sawdey et al., 2019; Creamer et al., 2021). One study showed that adolescents having one best friend who smoked increased the likelihood of initiating smoking by almost five times. Moreover, adolescents who had a higher percentage of friends who smoked were four times more likely to initiate smoking at a younger age than their peers (Khuder et al., 2008). However, very early initiation of smoking may be driven more by family and personal attraction to smoking than by peer influence (Loan et al., 2021). Cigarette and e-cigarette use are associated with similar factors, however, youth who use both types of products may have more risk factors compared to those who report to be single product users (Sawdey et al., 2019).

Research among adults’ motives for tobacco show a stark contrast with motives for alcohol or cannabis use, since it is less strongly associated with coping with negative emotions and primarily seen as habitual and automatic behavior that is largely driven by withdrawal cues (which can be experienced as negative emotions as well), because of its highly addictive nature (Cooper et al., 2015). Adolescents’ motives for smoking, however, can be centered around themes such as relaxation/pleasure, friends’ behavior and attitudes and the image of smoking (e.g., smokers are more popular, smoking is cool; Stanton et al., 1993). Additionally, in the aforementioned qualitive study among Dutch adolescents (Tabor, 2020), self-focused avoidance motives such as addiction and stress-related motives were more often mentioned for smoking than for alcohol and cannabis use (Tabor, 2020).

The most recent body of research on adolescents’ motivations for smoking mainly focusses on the use of e-cigarettes, because the novelty of this phenomenon. A survey among Mexican middle-school students showed that the most common reason for using e-cigarettes was curiosity in trying this substance (Zavala-Arciniega et al., 2019). This is consistent with other studies that show that the availability of flavors and the belief that the taste is better compared to regular cigarettes were important reasons for adolescents to start vaping (Sussan et al., 2017; Temple et al., 2017). Other reasons that were reported, were associated with the specific characteristics of vaping, such as that it was allowed in places where smoking is prohibited, that it helped in smoking fewer cigarettes or in quitting smoking altogether (Temple et al., 2017; Zavala-Arciniega et al., 2019). Specific to vaping is also the perception among adolescents that it is less harmful compared to regular cigarettes and that it can serve as a healthier alternative to smoking (Sussan et al., 2017). As is the case with alcohol, adolescents may also be more susceptible to the influence of advertisements and glamorization of vaping by celebrities (Sussan et al., 2017). In addition, just as smoking of regular cigarettes in movies and on television was found to encourage initiation of smoking in adolescents (Dal Cin et al., 2008), the role of the media in making smoking look cool is likely to have played a role in the rapid uptake of e-cigarettes in adolescents. Smoking tobacco among youth may be seen as one of the more acceptable forms of risk behaviors, because it does not have direct intoxicating effects and the health consequences are only visible at the long term. However, nicotine is highly addictive and smoking in public places has become less socially acceptable in Western societies. In conclusion, adolescents’ motives for smoking can be centered mostly around self-focused approach motives (e.g., experimenting) and social motives (e.g., smoking is seen as cool/tough).

### Current descriptions of adolescents’ motivations for engaging in cannabis use

It is important to also consider motivations for youth cannabis use, since cannabis is illegal in most countries, yet it is the most used illicit drug among youth. Youth can also develop a cannabis use disorder (Defoe et al., 2019). For example, in the Netherlands, cannabis outscores alcohol and tobacco, as the most diagnosed substance use disorder among youth (Braet and Bögels, 2014), and in the United States, it is the most common drug for which youth seek treatment (Johnston et al., 2015). However, unlike other substances such as alcohol and tobacco, cannabis has been used to treat medical conditions (Cohen et al., 2019), and thus especially medical cannabis use (versus recreational cannabis us) is currently being legalized to a larger extent. All these unique attributes of cannabis can thus have a different impact on motivations for cannabis use versus alcohol and tobacco use.

The predictors of youth cannabis use appear to be similar to alcohol and tobacco youth use. Both old and more recent (meta-analytical) reviews on predictors of youth cannabis use have consistently shown that demographic (lower socioeconomic status, male sex), personality (sensation seeking) and social factors (peers’ cannabis use, parent–child relationships problems) predict youth cannabis use (Guxens et al., 2007; Kirst et al., 2014). For example, in one longitudinal study, peer cannabis use predicted adolescent cannabis over three waves, and these cascading links predicted cannabis use disorder in emerging adulthood (ages 18–20; Defoe et al., 2019). As for media effects, unlike multiple studies on the effects of adolescents’ media exposure to alcohol and tobacco use (e.g., Sargent et al., 2005; Dal Cin et al., 2008; Curtis et al., 2018), similar studies on cannabis use are lacking and are primarily limited to a few studies on cannabis advertisements in the media. For example, Fa recent study showed that adolescents’ exposure to cannabis marketing in states in the United States with legalized cannabis laws was associated with recent cannabis use (Whitehill et al., 2020).

Although there is a plethora of studies on the predictors of cannabis use, research on the motivations of cannabis use has lagged behind, especially compared to the relatively vast research on the motivations for alcohol use (*cf* Cooper et al., 2015). Nevertheless, the assessment of cannabis use motivations has been inspired by the motivations for alcohol use (see, e.g., Simons et al., 1998). However, while there can be overlapping motivations for alcohol and cannabis use, there might also be some unique motivations per substance. For example, the Marijuana Motives Measure (MMM; Simons et al., 1998), was inspired by Cooper’s (1994) original four factor alcohol motivational model (i.e., Coping, Conformity, Enhancement, Social). However, the “expanded experiential awareness” (i.e., altered perceptions) motive needed to be additionally included in the Marijuana Motives Measure to capture the unique psychedelic effects produced by cannabis use (Simons et al., 1998).

Of note is that after the development of the Marijuana Motives Measure Lee et al. (2007, 2009) followed-up by developing the Comprehensive Marijuana Motives Questionnaire (CMMQ). The Comprehensive Marijuana Motives Questionnaire was created from self-generated reasons for cannabis use that were reported by emerging adults (*N* = 634; 57.9% female) who were in-coming college students (i.e., recent high-school graduates) in the United States. From a total of 19 motives that were mentioned from the open-ended questions, a total of 12 subscales (“motivations”) were identified *via* a factor analysis. The 12 subscales were: (1) Enjoyment, (2) Conformity, (3) Coping, (4) Experimentation, (5) Boredom, (6) Alcohol, (7) Celebration, (8) Altered Perception, (9) Social Anxiety, (10) Relative Low Risk, (11) Sleep/Rest, and (12) Availability (Lee et al., 2007, 2009). Follow-up analyses showed that experimentation and availability motives were uniquely associated with lower levels of cannabis use. However, the enjoyment, boredom, altered perception, relative low-risk, and sleep/rest motives were uniquely associated with higher levels of cannabis use (Lee et al., 2009). After controlling for levels of use, the enjoyment motive was associated with fewer negative consequences while using cannabis or as a result of cannabis use (e.g., “Not able to do your homework or study for a test”), whereas coping and sleep/rest were associated with more negative consequences (Lee et al., 2009). Of note, is that the sample consisted of college students (emerging adults).

The only adolescent (ages 13–16) study we are aware of that was based on normative (non-high risk or non-clinical) individuals, is our aforementioned qualitive study among Dutch adolescents (Defoe et al., 2016; Lochs, 2020; Tabor, 2020). This study found that for cannabis use, most frequently mentioned motives were acting cool/tough, enjoyment (e.g., for “the feeling), “belonging” and “experimentation” (Tabor, 2020). Interestingly, these motives overlap with the above-mentioned motives that were reported in Lee et al. (2007), besides for the “acting cool/tough” motive. However, in our study, we did not investigate whether the reported motives were related to cannabis use and/or cannabis-use related problems. As for the literature on *high-risk* adolescents, of note is a recent study that used the CMMQ contained 252 adolescents attending high-school (9th–11th graders), albeit they were heavy cannabis-users who were enrolled in motivational enhancement/cognitive behavioral intervention (Blevins et al., 2016). That study reported that particularly the coping motive (i.e., using cannabis to forget one’s problems, or due to feeling depressed, or to escape from one’s life) was associated with more cannabis-related problems, lower levels of self-efficacy for avoiding cannabis use, and higher levels of internalizing and externalizing symptoms (Blevins et al., 2016).

In sum, when youth (1st year college students) in the United States are asked about their reasons to engage in cannabis use, the top 3 motives reported are *enjoyment/fun, conformity,* and *experimentation,* whereas for heavy-cannabis youth users, the top 3 motives that are most commonly endorsed are *enjoyment, availability, and sleep* (Blevins et al., 2016). However, Dutch youth reported that their top 3 motives are *to act tough or cool, for enjoyment, and belonging* (Tabor, 2020). Thus, interestingly, the primary motive for youth from the United States (enjoyment) is very different than youth from Netherlands (being cool or tough). Finally, based on at least one adolescent study with an at-risk sample (i.e., heavy cannabis-using youth) in the United States, we can tentatively conclude that particularly the coping motive is associated with more (cannabis use-related) problems in adolescence (Blevins et al., 2016).

To conclude, it can be extrapolated from our review of the literature that adolescents’ quest for a “cool/tough identity” is important to understand their engagement in risk behavior, since in our study (Lochs, 2020; Tabor, 2020 see also Lee et al., 2007), adolescents mentioned being cool/tough as the primary motive for engagement in alcohol, tobacco, and cannabis use. This quest for a cool/tough identity could be tied with experimentation, which is also among the most-mentioned motives by normative adolescents to explain their cannabis use (Lee et al., 2007; Lochs, 2020; Tabor, 2020). Of note, although prior studies have shown that adolescents report experimentation among the most common motives for cannabis use in both the United States (Lee et al., 2007) and the Netherlands (Tabor, 2020), according to the above-described DNERM framework, it might be that youths with lower levels of self-control, are the ones who would find it more difficult to discontinue with substance use after the experimentation phase is over (*cf* Defoe, 2021).

#### More recent positive risk-taking research

Recently there has been growing recognition that some risk-taking is adaptive for adolescents who are seeking to define their identities and learn about their place in the world. Adolescents are poised to gain this understanding through their increasing ability to learn from experience and gain control over their behavior (*cf* Defoe and Romer, 2022). There is also a growing recognition that the dominant meaning of risk-taking in the developmental literature follows the lay usage of behavior that risks harmful outcomes, such as drug use, unprotected sex, and distracted driving. However, adolescents may not view risk behavior from the same perspective as adults (Defoe and Romer, 2022). Namely, as noted above, Skaar (2021) found that educational risk behaviors (e.g., taking challenging classes) were the most mentioned by youth as risk behaviors, followed by “drug and alcohol use” and “trying something new.” Related to this, Duell and Steinberg, (2021) recently presented an approach that focuses on adaptive forms of risk taking, such as developing a new skill, that need to be encouraged despite the prospect of challenges that such learning may require.

#### Labeling theory and adolescent risk-taking

As mentioned above, adolescents are often labeled as the stereotypical risk taker, in society and in scientific research. This characterization of adolescents implies that it is normal or expected that adolescents engage in heightened risks compared to other age groups. This idea that adolescents are the stereotypical risk-takers, often carries a negative connotation. For example, it is often associated with the maladaptive types of risk taking (e.g., binge drinking or engaging in delinquency). However, adolescents engage in adaptive risks too, although this is far less investigated (Duell and Steinberg, 2021; Defoe and Romer, 2022). Moreover, laboratory studies on age differences in risky decision-making show that adolescents do not always engage in heightened risk-taking (Defoe et al., 2015). Recent theories (see below) and reviews (Willoughby et al., 2021) suggest that adolescents might only be overrepresented in some types of risk behaviors (e.g., minor delinquency), whereas it is emerging adults who are over-represented in other types of risk behaviors (e.g., substance use). In sum, adolescents are not the only “stereotypical” risk-takers. But what consequences does labeling them as such have on their levels of engagement in risk-taking? This question appears to have not yet been addressed in research, and hence below we start the conversation about possible consequences of such labeling in the context of labeling theory (Becker, 1963).

Labeling theory originated in the sociology discipline (e.g., Becker, 1963). In criminology, this theory is among the primary classical theories used to explain criminal behavior (Murray and Farrington, 2014; Bernburg, 2019). Originally, one of the main questions labeling theory sought to answer was *what are the effects of official labeling (e.g., arrest and conviction) on subsequent (criminal) behavior?* (Murray and Farrington, 2014). This is exactly the question we explore in this part of the review. *Namely, what is the effect of the historically ingrained label “stereotypical risk-takers” that has been associated with the adolescent period on subsequent adolescent risk behavior?* By exploring this question, we aim to start a conversation in the field of psychology, considering that labeling theory is not commonly used among psychologists who study criminal behavior or risk behavior more generally. Of note is that there has been some attention by sociologists (Scheff, 1966) on labeling of mental illness which is an inherent topic of the fields of psychology and psychiatry. Namely, it has been argued whether when an individual is labeled as “mentally ill,” these individuals adapt their behaviors to fulfill the expectations of such a label (Pasman, 2011; Scheff, 1966). Here we apply the same reasoning to the label of stereotypical “risk-taker.”

Extrapolating from labeling theory, it is particularly the “deviant self-concept” that is likely to mediate the link between deviant labeling (e.g., “juvenile delinquent”) and deviant behaviors (Murray and Farrington, 2014). This is an important assertion when considering the impact labeling might have during the adolescent period, when individuals are still exploring their identity. Extrapolating from the symbolic interactionist theory (Mead and Schubert, 1934)—which was inspired by labeling theory—it is conceivable that *“when adults label a youth a “troublemaker,” the youth may come to see himself as a troublemaker, eventually adopting the identity as a troublemaker”* (p. 14–15, Matsueda, 2014). This association begs the question whether labeling adolescents as stereotypical risk-takers contributes to them identifying as such, which then leads to higher levels of risk behaviors in the future.

To the best of our knowledge, the above-described mediation question which has far-reaching implications has not been empirically investigated within the context of risk-taking. More specifically, the question as to *whether labeling adolescents as stereotypical risk takers fosters a risk-taking identity in adolescents, which in turn leads to higher levels of adolescent risk-taking* has been unexplored. In fact, although in criminology there is strong evidence that criminal labeling predicts more criminal behavior (see Murray and Farrington, 2014), empirical longitudinal tests of a similar mediation *via* “criminal identity” is also difficult to retrieve in the criminological literature (see Murray and Farrington, 2014; Bernburg, 2019). Nevertheless, a groundbreaking longitudinal study based on data on adolescent males from the National Youth Survey, showed that parental labeling of their sons as rule-breakers predicted subsequent adolescent delinquency *via* their sons’ own views of themselves (reflected appraisals of self) as rule-breakers (Matsueda, 1992). Additionally, a more recent retrospective study corroborated these results by showing that adolescent’s own delinquent self-views (delinquent identity) mediated the link between “their reflected appraisals of delinquency by others” (parents and friends) and “future adult delinquency” (Walters, 2016). In Walters (2016), “reflected appraisals of delinquency by others” referred to retrospective accounts of the participant’s interpretation of the actual appraisals of their parents and peers. Alternatively, other scholars have suggested that deviant peer affiliation might also be an important factor that mediates the link between such deviant labeling and deviant behavior (for an overview, see: Bernburg, 2019). This is another relevant hypothesis, particularly for adolescence, as this period has been conceptualized as being associated with heightened susceptibility to deviant peer influence (Brechwald and Prinstein, 2011).

Of course, similar to how multiple mediators might be at play in the link between such deviant labeling and future deviant behavior, multiple moderators might be at play as well. That is, labeling might not affect all youth to a similar extent. Or as eloquently put: *“Of course, different youths respond to negative labeling in different ways—sometimes actively resisting with aggression, sometimes fleeing, and sometimes surrendering”* (p. 20, Matsueda, 2014). For example, boys might be more susceptible to the “stereotypical risk taker” label, due to social or cultural norms that imply that certain deviant behaviors are more acceptable for males compared to females. Finally, of note is that we do not posit that labeling of adolescents as stereotypical risk-takers is the sole cause of risk behaviors during the youth period, but it warrants research attention to confirm whether it is a contributing factor.

## Discussion and conclusion

Risk behaviors such as substance use typically show accelerated increases during adolescence. In fact, adolescents have virtually always been considered as the “stereotypical risk-takers” in science, but also in the mainstream media, fictional literature and in everyday life. However, adolescents’ own perspectives for engaging in risk-taking have been largely neglected in research on adolescent risk-taking, and increasing research suggests that adolescents do not always engage in “heightened” risk-taking. Hence in this paper, we argued that if it is the intention to achieve a comprehensive understanding of risk-taking during adolescence, then the perspectives and experiences of the adolescent need to be identified in research. This is also in line with the participatory approach advocated by international children’s rights standards. Hence, using a culturally sensitive approach, we summarized the few empirical studies on adolescents’ own motivations for engaging in substance use (cannabis use, alcohol use, and smoking). We found that being cool/tough, enjoyment (the taste of the substance or the feeling it gives), belonging (fitting in/impressing others), having fun, experimenting and coping (e.g., stress reduction) were frequently mentioned by youth as motives for substance use. Addiction was additionally mentioned for smoking. Interestingly, in our study among Dutch adolescents (Lochs, 2020; Tabor, 2020), the motive that was mentioned the most “stoer doen” (acting cool or tough) is not a common theme in adolescent risk-taking research (but it was mentioned to a lesser extent in the study by Lee et al., 2007). It could be of added value to investigate whether “cool” or “tough” identity motive predicts risk-taking behavior in modern times among *adolescents*, in addition to more currently explored factors such as sensation seeking and peer influence. Identity formation is an important task for adolescents and thus it would be meaningful for research to investigate the implications that comes with the drive of adolescents to acquire a “cool/tough” identity. The above-described findings on youth motivations to engage in substance use generally support the *Developmental Neuro-Ecological Risk-taking Model* (DNERM; Defoe, 2021) and the *Life-span Wisdom Model* that suggest that adolescents’ motivations to engage in substance use, include experimentation, exploration, and sensation seeking. These conclusions all run counter to the stereotype of adolescents engaging in risk-taking especially due to “storm and stress.” We further conclude that although the quantity and types of risk behaviors might differ across countries (e.g., United States and Europe), the frequently mentioned motives (i.e., to be cool/tough, enjoyment, belonging, having fun, experimenting, coping; and additionally, addiction for smoking) to engage in substance-use appear to be similar across cultures. Still, of note is that unlike other youth, Dutch youth frequently mentioned “stoer doen” as a motive for engaging in substance use, which can be translated as “acting cool” or “acting tough” in English. Although the current literature suggests that “rebelliousness/roughness” is a component of the concept of “coolness” (see Dar-Nimrod et al., 2012, 2018), we did not retrieve literature from other countries (besides the Netherlands) that referred to “acting tough” as a motive for substance use (but see Lee et al., 2007, in which a minority of college students mentioned “to be cool/to feel cool” as a motive for cannabis use). But then again, the literature on self-generated motivations of youth for risk-taking is still in its infancy, and if more studies were to assess self-generated motives, then adolescents might more often mention some form of “coolness” as a motive. Self-generated motives can be assessed by applying straightforward qualitative methods (see, e.g., Skaar, 2021). All in all, this observation pertaining to the “being cool/tough” motive, perhaps suggests that although motives for risk behaviors such as substance use might generally be the same across countries, still unique motives might be encountered across countries (cultures), which could also be tied to differences in linguistics. Clearly, we still have a lot to learn about self-generated motives by adolescents, especially since adolescents have a greater chance of becoming dependent on substances (Chambers et al., 2003).

Finally, we made a case for why nuance is warranted when labeling adolescents as stereotypical risk-takers. In doing so, we applied the labeling theory (Becker, 1963) in the context of adolescent risk-taking research. Extrapolating from the sociological/criminological literature on labeling, we put forward that labeling adolescents as stereotypical risk takers may instigate a risk-taking identity in adolescents and/or motivate adolescents to associate with risk-taking peers, and both could in turn lead to adolescent risk-taking. Besides these individual and social mediators, moderators could also be at play as all adolescents might not be equally susceptible to labeling effects. Research on labeling within the context of adolescent risk-taking is needed to confirm these speculations, and that research could further benefit from taking the adolescent’s motivations for engaging in risk-taking into account.

It is of importance to further cross-nationally investigate the behaviors that adolescents and young adults themselves consider to be risky and their motivations for engaging in these behaviors. This could more thoroughly explore the idea that young adults may engage in more extensive risk behaviors such as substance use, because they have the legal age to do so. This may also give further clarification on the influence that peers may have, which is generally considered stronger in early adolescence compared to late adolescence and young adulthood, and on the relation between peer influence and motivations. In other words, is heightened peer influence related to the desire to act “cool” or “tough”? Such research could have both extensive policy and clinical implications. It may further shape the image that adults have of adolescents. For example, not automatically labeling adolescents as the typical risk-takers, and thereby directing the view to more acceptable forms of risk behavior, such as educational risks and activism, that may have positive outcomes for both adolescents and society. Adolescent’s perspective on all this ought to be acknowledged, as that respects the important right of young people to be heard in accordance with the international children’s right standards.

## Author contributions

ID developed the study concept and outline, co-wrote the paper, and provided critical revisions thereafter. DR and SR co-wrote the paper and provided critical revisions. All authors contributed to the article and approved the submitted version.

## Conflict of interest

The authors declare that the research was conducted in the absence of any commercial or financial relationships that could be construed as a potential conflict of interest.

## Publisher’s note

All claims expressed in this article are solely those of the authors and do not necessarily represent those of their affiliated organizations, or those of the publisher, the editors and the reviewers. Any product that may be evaluated in this article, or claim that may be made by its manufacturer, is not guaranteed or endorsed by the publisher.
